# DeepForgeryNet: a hybrid CNN–LSTM and transfer learning framework for robust image forgery and deepfake detection

**DOI:** 10.3389/frai.2026.1810912

**Published:** 2026-05-08

**Authors:** Aarti Sardhara, Vipul Vekariya, Ajeet Ram Pathak, Sital Dash

**Affiliations:** 1 Department of Computer Science and Engineering, Faculty of Engineering and Technology, Parul Institute of Engineering and Technology, Parul University, Waghodia, Gujarat, India; 2 Norwegian University of Science and Technology, Trondheim, Norway; 3 Vishwakarma University, Pune, Maharashtra, India

**Keywords:** cross-dataset generalization, deepfake analysis, digital image forensics, error level analysis, hybrid CNN–LSTM, image forgery detection, transfer learning

## Abstract

**Introduction:**

The increasingly lifelike nature of digitally manipulated images, as well as those generated by AI, presents significant problems for both media authenticity and digital trust. Various detection methods mostly depend on the visual content and thus, might miss the subtle forensic traces. This paper focuses on developing a powerful detection tool that leverages both artifact-level and contextual inconsistencies.

**Methods:**

In this work, we introduce DeepForgeryNet, an artifact-savvy deep learning model that combines Error Level Analysis, based preprocessing with a hybrid CNN, LSTM network. The preprocessing step focuses on exposing changes in compression and edges, whereas the CNN, LSTM model hybridly captures the spatial and contextual information. The model is trained end-to-end on publicly available benchmark datasets stratifying the data splits.

**Results:**

The approach taken by the method proposed yielded the following results: accuracy at 95.1%, precision at 94.6%, recall at 94.2%, F1-score at 94.4%, and AUC at 0.98. It surpassed a baseline CNN and transformer models, especially in recall. Stability of generalization with accuracy over 92% was observed in cross-dataset experiments.

**Discussion:**

Such results serve as evidence that the integration of artifact-aware pre-processing with spatial-contextual feature learning can further enhance the reliability of detection of forgery. Even though there are still problems in the detection of very small manipulations and/or when heavily compressed, this work lays the groundwork for a new generation of digital media verification that is trustworthy.

## 1 Introduction

### 1.1 Background and motivation

Digital images are increasingly becoming the information and evidence sources in such areas as journalism, social media, healthcare, law enforcement, and scientific documentation. Therefore, the legitimacy of these pictures is highly linked with the trust of the public and their decision-making (Tolosana et al., 2020; Verdoliva, 2020). On the other hand, with the swift progress of image editing software and generative models, the possibility of making very realistic image manipulations has been democratized and made very easy (Barni et al., 2021a; Rössler et al., 2020; Tolosana et al., 2020; Verdoliva, 2020). In consequence, image verification has become a serious issue.

Exacerbate misinformation, defamation, and compromised forensic investigations are just a few of the consequences of altered images (Barni et al., 2021a; Cozzolino et al., 2021b; Verdoliva, 2020). The appearance of deepfakes and AI-generated visual content has made these dangers even greater by allowing the creation of convincing synthetic content (Mirsky and Lee, 2021; Rössler et al., 2020; Tolosana et al., 2020). Such situations stress the importance of creating trustworthy and large-scale image forgery detection methods without delay.

Digital image forensics focuses on identifying visual content that shows signs of tampering and inconsistencies (Barni et al., 2021a; Verdoliva, 2020). Strong forgery detection has become a critical element of the digital realm in order to keep digital trust and to be able to rely on these tools in investigative and legal processes (Cozzolino et al., 2021b; Lu and Ebrahimi, 2024).

### 1.2 Image forgery in the era of deep learning

Image forgery is a broad term that describes all sorts of manipulations including copy, move, splicing, object removal, and retouching, basically altering visual content while keeping it perceptually plausible (Nguyen et al., 2021; Verdoliva, 2020). Naturally, traditional editing methods have now been supported, and at times even surpassed, by deep learning-based synthesis methods. In fact, generative models such as GANs and autoencoder-based architectures have a very impressive outcome and can generate photo-realistic images that are almost indistinguishable from real ones (Bondi et al., 2023; Rössler et al., 2020; Tolosana et al., 2020).

Obviously, these kinds of technological advancements can be useful in media production, virtual reality, and even data augmentation, but at the same time, they can be misused in a very harmful way (Mirsky and Lee, 2021; Tolosana et al., 2020). The generation of deepfakes, impersonation, and fabricated visual evidence are the main difficulties of forensic analysis (Dang et al., 2022; Rössler et al., 2020; Tolosana et al., 2020). As synthetic content is gettingmore and more lifelike, the detection systems are required to look for the slightest defects (Bondi et al., 2023; Rössler et al., 2020).

### 1.3 Limitations of existing detection approaches

Forgery detection techniques can be broadly divided into active and passive (Barni et al., 2021a; Verdoliva, 2020). Examples of active techniques are watermarking and digital signatures, which require prior embedding of authentication information and hence cannot be used in most real-world situations (Barni et al., 2021a; Cozzolino et al., 2021b). Passive methods identify intrinsic cues, such as noise statistics, compression artifacts, and lighting inconsistencies, but most of them depend on handcrafted features that may not be very generalizable (Nguyen et al., 2021; Verdoliva, 2020).

Among different deep learning approaches, convolutional neural networks (CNNs) have helped improve the detection performance by enabling automatic feature learning (Bappy et al., 2021; Bondi et al., 2023; Rössler et al., 2020). That said, there are still several issues that need to be addressed. For instance, a large number of models take mainly RGB inputs and do not specifically focus on artifacts related to manipulation (Bondi et al., 2023; Rössler et al., 2020). Error Level Analysis (ELA) is one such technique that reveals compression inconsistencies; however, it is often used as a standalone method instead of being integrated into end-to-end learning pipelines (Barni et al., 2021a; Verdoliva, 2020).

Besides, the majority of deep models only extract spatial features and barely consider the contextual relationships between features (Bappy et al., 2021; Saikia et al., 2022). This limits their ability to detect minor or spatially dispersed manipulations. Moreover, models with high performance usually cost a lot in terms of computation, which makes it difficult to use them in real-time or resource-limited settings (Gong and Li, 2024; Xi and Chen, 2025).

### 1.4 Research gap

Although considerable progress has been made in the field of deep learning-based image forgery detection, there are still some issues that need to be addressed. A large proportion of the existing methods are based on RGB image inputs and devote most of the attention toward spatial feature extraction, thus ignoring artifact-level traces that are inherently related to the manipulation process (Bondi et al., 2023; Rössler et al., 2020). Moreover, steps are not consistently taken to utilize manipulation-induced compression pattern discrepancies as one of the features within end-to-end learning frameworks (Verdoliva, 2020).

Error Level Analysis (ELA) stands out as a well-known forensic method for identifying compression anomalies, particularly (Verdoliva, 2020). Nonetheless, it most frequently serves as an independent method without being combined with deep learning techniques in a structured manner (Barni et al., 2021a). Thus, what remains to be explored is the potential of combining artifact-level analysis and deep feature translation as a complementary pair. Otherwise, most deep models mainly focus on spatially local features and barely consider the contextual relationships among feature regions (Bappy et al., 2021; Saikia et al., 2022). The research on sequential modeling methods that can capture the dependencies between different regions is quite limited in the field of image-level forgery detection.

Moreover, the issue of generalizability and practical applicability is still unresolved. A model that scores very high on a particular dataset may prove less robust when tested under cross-dataset conditions due to dataset bias as well as variations in compression, resolution, and manipulation techniques (Gupta et al., 2024; Mirsky and Lee, 2021). Besides that, detection method performance is not always judged jointly with computational efficiency, which is a very important factor when deploying real-world forensic tools (Xi and Chen, 2025). These limitations suggest that a unified framework is needed that combines artifact-aware preprocessing, robust deep feature learning, contextual modeling, and efficiency-aware design tightly packed in one detection pipeline.

### 1.5 Novelty and key innovations

Hybrid CNN, LSTM architectures for forgery and deepfake detection have been explored in some of the earlier works. However, the DeepForgeryNet presented in this paper has several distinguishing features that set it apart from the existing methods. Firstly, contrary to the traditional approaches that mostly use RGB inputs or disjoint preprocessing steps, this paper presents a unified artifact-aware preprocessing pipeline that combines Error Level Analysis (ELA), Bayesian denoising, PSNR-based quality validation, and edge enhancement in a well-ordered and sequential manner. This mix allows to explicitly highlight forensic cues specific to manipulation before feature learning, which is not a common feature of prior CNN, LSTM frameworks. Secondly, our framework changes the perspective of image-level forgery detection to a spatial, contextual learning one by transforming the CNN feature maps into structured sequences that can be modeled by LSTM. It is true that LSTM models are heavily used for video analysis, but their ability to capture inter-regional dependencies within single images is still a less developed area. Third, this paper views cross-dataset generalization as a fundamental aspect in the design of the method. The model is thoroughly tested on different benchmark datasets that contain various types of manipulations and have different levels of compression, which proves that it is not only relying on dataset-specific features. Additionally, the model building pipeline is efficiency-driven to strike a good balance between detection results and computational cost. In contrast with numerous recent transformer-based methods that need massive computing power, the proposed method is able to produce accurate results with a relatively simple model, thus it is well suited for real forensic use cases. All in all, these results highlight DeepForgeryNet as a comprehensive conceptual design that combines artifact-level identification, deep feature extraction, contextual understanding, and the ability to generalize all in one integrated pipeline.

### 1.6 Contributions of this work

This paper presents DeepForgeryNet, a versatile tool that detects forged images and videos and deepfakes by combining artifact-aware preprocessing with hybrid deep learning. The main contributions of this research can be summarized as follows:

1. Single artifact-aware preprocessing pipeline: We develop a thorough preprocessing pipeline that integrates Error Level Analysis (ELA), Bayesian denoising, PSNR-based quality validation, and edge enhancement techniques giving a significantly strong localization of manipulation-related artifacts before the extraction of deep features. Unlike the methods relying solely on raw RGB inputs, this pipeline turns up the forensic clues to a level that they can be used for tampering detection with almost absolute certainty.
2. Hybrid spatial contextual modeling: Our proposal is a hybrid CNN-LSTM framework which not only captures spatially localized artifacts but also accounts for the contextual dependencies among feature regions in a joint manner. Via this scheme, the model is made capable of picturing manipulations that are inconspicuous and very much scattered over the space to the extent that even a CNN-only setup might not have recognized them.
3. Thorough multi-dataset evaluation: The proposed massively is put through the ringer in terms of several landmark datasets, e.g., CASIA V2.0, FaceForensics++, Celeb-DF, and DFDC, which involve different types of manipulations and compression levels. This kind of diverse testing protocol makes it possible to have a more authentic evaluation of the robustness and cross-domain generalization of the method.
4. Component-wise validation via ablation experiments: Each of the ablation tests demonstrates the extent to which various components contribute to the detector’s overall efficiency, as far as surface-level changes, noise removal, and sequence modeling are concerned. Therefore, it serves as a good empirical guideline for each unit.
5. Efficiency conscious design: The authors show possible ways of improving detection even with a slight increase in the parameter count, thus it remains very convincing that their method would work well if implemented in real forensic cases.

### 1.7 Research questions

To guide the empirical evaluation of the proposed framework, this study investigates the following research questions:

*RQ1:* Does incorporating artifact-aware preprocessing, including Error Level Analysis and denoising, improve forgery detection performance compared to using standard RGB inputs?

*RQ2:* Can combining spatial feature extraction with contextual modeling through a hybrid CNN–LSTM architecture enhance detection reliability relative to single-architecture models?

*RQ3:* How robust is the proposed framework when evaluated across datasets with differing manipulation techniques, compression levels, and visual characteristics?

*RQ4:* Can high detection performance be achieved while maintaining computational efficiency suitable for practical forensic applications?

### 1.8 Organization of the paper

The rest of the paper is organized as follows. Section 2 elaborates on the materials and the methodology proposed. Section 3 details the experimental results and performance evaluation. Section 4 interprets the results and compares the proposed approach with the existing state-of-the-art methods. Last, Section 5 summarizes the paper and offers the directions for future research.

## 2 Related work

The field of image forgery and deepfake detection has seen a number of significant breakthroughs thanks to the application of deep learning. Researchers have primarily focused their work on a few different themes that include CNN-based detection methods, utilizing transfer learning and domain adaptation, employing hybrid sequential models, applying transformer-based approaches, artifact-aware preprocessing, and investigations related to robustness and interpretability.

### 2.1 CNN-based forgery detection

One of the main reasons why convolutional neural networks (CNNs) are at the core of various forgery detection systems is their capability to pick up on discriminative spatial features. Deep models like ResNet, VGG and Xception have been extensively used and demonstrated that the localization of manipulation traces and visual inconsistencies, still pretrained models show a very strong performance (Al-Dulaimi and Kurnaz, 2024; Kurnaz, 2024; Masood et al., 2023). Their successful performance has been evidenced through repeated experiments where they have outperformed handcrafted forensic descriptors in splicing and copy, move detection tasks (Heo et al., 2023; Khalid et al., 2022). On the other hand, CNN-based methods primarily focus on extracting semantic content and, thus, might fail to detect compression-related traces. For this reason, a number of researchers have proposed the integration of residual representations and noise-based features that help to uncover slight manipulation artifacts (Coccomini et al., 2022b; Frank et al., 2020; Wang et al., 2020). The most advanced methods simultaneously feed CNNs with artifact-enhanced inputs, which results in better resilience against various post-processing operations (Barni et al., 2022; Durall et al., 2020a).

### 2.2 Transfer learning and domain adaptation

Transfer learning can be a viable approach when forensic datasets are small. Usually, models that start with pretrained weights reach convergence faster and yield higher accuracy than ones trained from scratch (Coccomini et al., 2022c; Mittal et al., 2021; Zhou et al., 2021). Besides, domain adaptation methods make it possible to lessen the bias in datasets and enhance generalization to unseen datasets (Chen et al., 2023; Zhang et al., 2021). However, transfer learning by itself has no direct focus on forgery artifacts and can pick up biases from source domains. As a result, newly published research advises integrating transfer learning with artifact-aware inputs to achieve reliable performance (Khalid et al., 2021a; Trinh et al., 2021).

### 2.3 Hybrid CNN–LSTM and sequential models

Hybrid CNN-LSTM architectures combine the extraction of spatial features with the modeling of sequential dependencies. Such models are intensively applied in the detection of video deepfakes and have been re-purposed for contextual modeling at the image level by handling feature maps as sequences (Gragnaniello et al., 2021a; Gragnaniello et al., 2021b; Mandelli et al., 2022). Through sequential modeling, detectors become capable of recognizing correlation between different regions as well as spotting manipulation traces that are spread out. Moreover, the application of optical-flow guidance and attention mechanisms in variants has helped to further enhance the sensitivity to forgery patterns (Bonettini et al., 2021; Chen et al., 2022; Coccomini et al., 2022a). Besides, there have been proposals for lightweight CNN-LSTM ensembles suitable for real-time detection scenarios (Cozzolino et al., 2021a; Zhu et al., 2023).

### 2.4 Transformer-based approaches

Vision Transformer and hybrid CNN-Transformer models have attracted the attention of researchers for their capability to model long-range dependencies. Using self-attention mechanisms, detectors learn to concentrate on informative regions leading to the improvement of manipulation localization (Barni et al., 2021b; Gragnaniello et al., 2021c; Vyas et al., 2024). Patch-based transformers and hierarchical attention networks have been shown to perform on a par with as well as outperform deepfake datasets (Durall et al., 2020b; Zhang et al., 2021). Nevertheless, transformer-based architectures in general necessitate larger datasets and a higher computational cost, thus possibly being a limitation for their deployment in real-world forensic scenarios (Khalid et al., 2021b; Trinh et al., 2021; Wang et al., 2020).

### 2.5 Artifact-aware preprocessing

Historically, the study at the artifact level was and is one of the main directions in digital forensics. Various methods including Error Level Analysis (ELA), investigation of inconsistencies in compression, and extraction of noise residuals reveal traces of tampering even before learning of features (Durall et al., 2020a; Frank et al., 2020; Gragnaniello et al., 2021a). It has been evidenced by the latest research that combining artifact-aware preprocessing with deep learning results in better resistance to compression and noise distortions (Bonettini et al., 2021; Gragnaniello et al., 2021b; Mandelli et al., 2022). Feature extraction can be made more robust with the help of Bayesian denoising and quality-guided preprocessing (Durall et al., 2020b).

### 2.6 Generalization, robustness, and interpretability

Generalization across datasets remains a major challenge in the field. Neural networks that get trained on a single database tend to deteriorate when they are tested on new data that differ in terms of the distribution (Chen et al., 2022; Frank et al., 2020; Khalid et al., 2021b). Training on multiple datasets and utilizing domain generalization techniques are the ways to significantly reduce this problem (Barni et al., 2022; Coccomini et al., 2022a; Zhu et al., 2023).

Interpretability plays an ever bigger role in forensic use-cases. Through attention visualization and localization techniques, transparency can be improved and human-in-the-loop validation becomes possible (Cozzolino et al., 2021a; Masood et al., 2023; Tolosana et al., 2020). In addition, explainable AI mechanisms play a significant role in the development of forensic trust (Barni et al., 2021b; Gragnaniello et al., 2021c; Wang et al., 2023). Robustness, explainability, and scalability were highlighted in the recent literature reviews as major unresolved issues in deepfake detection research (Chen et al., 2023; Frank et al., 2020; Yan et al., 2023).

### 2.7 Synthesis of related work

One of the most striking examples of recent breakthroughs is the role deep learning has play in enhancing the detection of image forgery and deepfake. In fact, despite significant progress, the current methods still mainly lack a holistic solution and mainly focus on isolated strengths (cornerstones) of the methodologies (CNN). CNN-based models offer excellent spatial feature learning ability. However, they still largely fail to rely on manipulation-specific artifact cues (Coccomini et al., 2022b; Frank et al., 2020; Kurnaz, 2024; Masood et al., 2023). On the other hand, transfer learning helps to convergence and generalization, which are essential, but may also reinforce dataset biases if forgery traces are not the primary target (Coccomini et al., 2022c; Khalid et al., 2021a; Mittal et al., 2021; Trinh et al., 2021). Hybrid CNN-LSTM approaches enrich the model with a notion of context, which helps to capture the inter-regional dependencies. However, they are more often applied to video analysis and can pose a risk of increased computational complexity (Bonettini et al., 2021; Chen et al., 2022; Gragnaniello et al., 2021a; Mandelli et al., 2022). Transformer-based approaches extend the ability of the model to capture long-range dependencies and even localization while generally being data-hungry and computationally expensive (Barni et al., 2021b; Trinh et al., 2021; Vyas et al., 2024; Wang et al., 2020). Besides, artifact-aware preprocessing techniques, including Error Level Analysis and noise residual extraction, are directly concentrating on tampering signals and thus can improve robustness under compression. Still, their association with hybrid deep models is relatively scanty (Bonettini et al., 2021; Durall et al., 2020a; Gragnaniello et al., 2021a; Gragnaniello et al., 2021b). In addition, cross-dataset robustness and interpretability still remain to be significant challenges because many detectors tend to deteriorate under distribution shifts and continue to act as black-box systems (Chen et al., 2022; Cozzolino et al., 2021a; Frank et al., 2020; Masood et al., 2023). Hence, as a coherent combined effort, the existing studies widely agree with that a single framework has to be capable of synergistically utilizing the combination of artifact-level cues, spatial, contextual modeling, and efficiency-aware design in order to achieve a robust and practical forgery detection. The key differences among recent detection approaches are summarized in Table 1, highlighting variations in artifact awareness, contextual modeling, and robustness.

**Table 1:** Comparison of recent image forgery and deepfake detection methods.

Study	Core method	Artifact awareness	Context modeling	Cross-dataset tested	Efficiency focus	Key limitation
Bondi et al. (Kurnaz, 2024)	CNN	Partial	No	Limited	Moderate	RGB-focused
Dang et al. (Al-Dulaimi and Kurnaz, 2024)	CNN	No	No	Yes	Moderate	Spatial-only
Cozzolino et al. (Coccomini et al., 2022b)	Domain Adaptation CNN	No	No	Yes	Moderate	Dataset bias persists
Kurnaz (Mittal et al., 2021)	CNN–LSTM + TL	No	Yes	Limited	Moderate	Weak artifact modeling
Bappy et al. (Gragnaniello et al., 2021a)	CNN–LSTM	No	Yes	Yes	Low	High complexity
Xi & Chen (Gragnaniello et al., 2021c)	Transformer	No	Yes	Limited	Low	Heavy computation
ELA-based methods (Gragnaniello et al., 2021a; Frank et al., 2020; Durall et al., 2020a)	Artifact + CNN	Yes	No	Limited	High	Weak contextual modeling
Domain generalization works (Frank et al., 2020; Khalid et al., 2021b; Chen et al., 2022)	Multi-dataset	No	No	Yes	Moderate	Limited artifact cues
XAI methods (Cozzolino et al., 2021a; Tolosana et al., 2020; Masood et al., 2023)	Explainable models	No	Partial	Limited	Moderate	Accuracy trade-offs

## 3 Methodology

**Figure 1:**
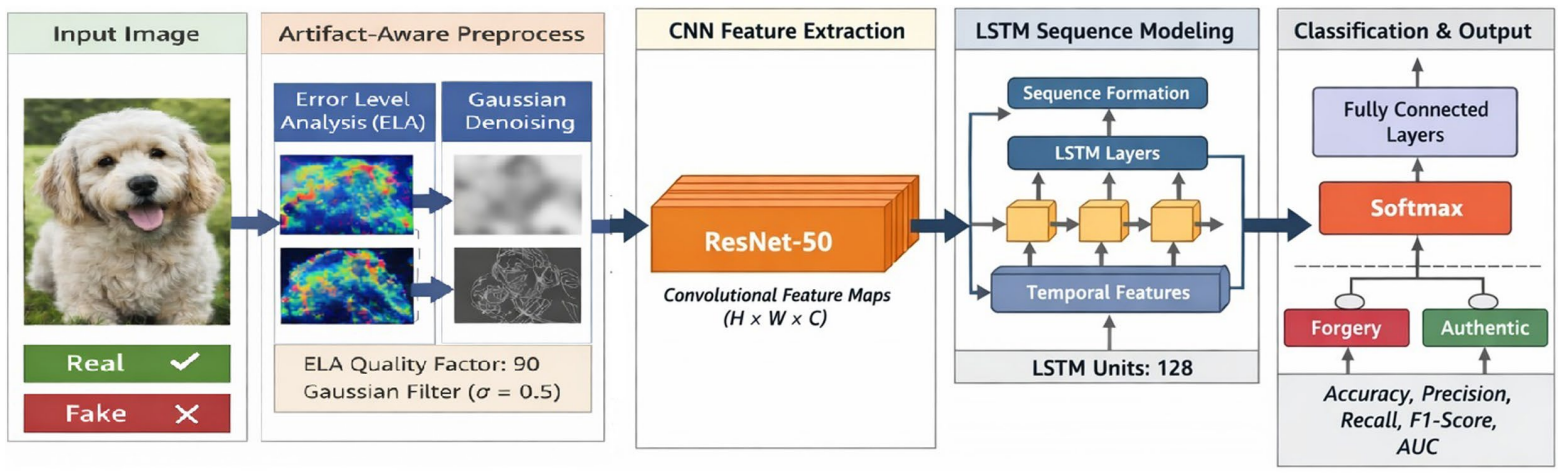
Overview of the DeepForgeryNet architecture, illustrating artifact-aware preprocessing, CNN feature extraction, LSTM-based contextual modeling, and final binary classification of images (original image created using OpenAI’s ChatGPT image generation capability through the model GPT-5.3, https://openai.com/index/gpt-5-3-instant/, with a full list of prompts found in the Supplementary material).

This paper puts forward an artifact-aware deep learning architecture DeepForgeryNet for reliable image forgery detection. The method is elaborated to jointly leverage semantic visual cues and manipulation-specific artifacts since the literature reveals that the RGB content alone may hide the forgeries of tampering traces. The architecture proposed marries artifact-enhancement preprocessing, deep spatial feature extraction, and contextual dependency modeling inside a single pipeline. As seen in Figure 1, the process starts with performing artifact-aware preprocessing to enhance the compression inconsistencies and boundary irregularities, then continues with CNN-based feature learning and LSTM-based contextual modeling for forgery classification. Through such composition, the model is permitted to identify not only the local modification traces but also the relationships between different image regions, which was demonstrated to increase the robustness of the detection of forgery.

### 3.1 Dataset input and preprocessing

#### 3.1.1 Data input

To make sure it was an all-round assessment, the DeepForgeryNet framework proposed was subjected to the scrutiny of a number of publicly accessible benchmark datasets which are commonly used in the research of image forgery and deepfake detection. These datasets taken together offer diverse types of manipulations, different compression levels, and real-world scenarios. Only the samples that were allowed for use by the respective datasets were considered.

##### 3.1.1.1 FaceForensics++ (FF++)

FaceForensics++ is among the datasets used most extensively for the detection of deepfakes. It features both unaltered and manipulated facial images that have been created by applying various facial manipulation techniques including DeepFakes, Face2Face, FaceSwap, and NeuralTextures. The dataset also offers images at different compression levels which can be used when assessing the robustness of a method.

- Approximate size: >1,000 original videos and over 500,000 extracted frames
- Manipulation types: Face swapping and reenactment
- Usage in this work: Frame-level forgery detection
- Availability: https://github.com/ondyari/FaceForensics (Access is granted for academic research upon request).

##### 3.1.1.2 CASIA V2.0 image tampering dataset (CASIA V2.0 v2.0)

CASIA V2.0 v2.0 is an important benchmark dataset widely used for the evaluation of general image forgery detection. It features genuine and tampered images with the use of splicing and copy-move techniques, showing various scenes and objects. This dataset also offers region-level tampering samples that are helpful for the analysis of artifacts.

- Total images: 12,614 (Authentic: 7,491, Tampered: 5,123)
- Manipulation types: Splicing and copy–move
- Usage in this work: General forgery detection
- Availability: https://www.kaggle.com/datasets/divg07/casia-20-image-tampering-detection-dataset (Publicly available for research).

##### 3.1.1.3 DeepFake detection challenge (DFDC)

DFDC dataset is one of the biggest deepfake datasets which was made available in order to test the performance of the detection algorithms in a real-life setting. It includes videos of different people, with different lighting conditions and compression levels, i.e., all factors that make a recording look more or less realistic in the world.

- Total videos: ~124,000
- Extracted frames: Millions (depending on sampling rate)
- Manipulation type: AI-generated face swaps
- Usage in this work: Cross-dataset generalization evaluation
- Availability: https://ai.facebook.com/datasets/dfdc (Publicly available for research).

The benchmark datasets used for training and evaluation, along with their key characteristics, are summarized in Table 2.

**Table 2:** Description of benchmark datasets used for evaluation.

Dataset	Data type	Forgery/manipulation types	Samples	Purpose
CASIA V2.0 V2.0	Images	Copy–move, splicing	~12,600	Image forgery detection
FaceForensics++	Images/Frames	Face swapping, reenactment	~1.8 M frames	Deepfake detection
Celeb-DF	Images/Frames	High-quality deepfakes	~600	Robustness evaluation
DFDC	Images/Frames	Diverse deepfake methods	~100 k	Large-scale validation

#### 3.1.2 Preprocessing and artifact enhancement

**Figure 2:**
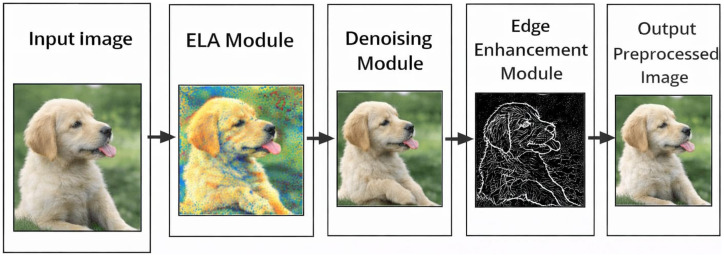
Preprocessing pipeline for artifact enhancement. The pipeline depicts sequential preprocessing operations, including Error Level Analysis, Bayesian denoising with PSNR-based assessment, and edge enhancement, applied to input images to emphasize manipulation-related artifacts prior to deep feature extraction (original image created using OpenAI’s ChatGPT image generation capability through the model GPT-5.3, https://openai.com/index/gpt-5-3-instant/, with a full list of prompts found in the Supplementary material).

Before feature extraction, an artifact-aware preprocessing stage was applied to both strengthen manipulation-related cues and keep semantic image content intact. Image forgeries usually make tiny inconsistencies in compression patterns, noise distribution, and boundary structures that cannot always be seen even in raw RGB space. So, to leave evidence of the manipulation and facilitate the detection by a learning model, the signal of forensics traces was the main focus in the preprocessing pipeline. The general artifact-enhancement work was demonstrated in Figure 2.

To begin with, Error Level Analysis (ELA) was utilized to discover compression inconsistencies. ELA essentially operates by re-saving an image at a predetermined quality level and then calculating pixel-wise differences between the original and re-saved images. Parts of the image that have been compressed several times, which is usually the case in tampered areas, generally show higher error levels. This phase of the work visually indicates potential manipulation areas and offers artifact-level signals to the model, as shown in Figure 2. After that, a denoising module was used to eliminate the noise introduced by the sensor and the environment while keeping the structural and textural information intact. The well-controlled denoising not only makes the signal more clear but also avoids the noise dominating the learned representations. More importantly, the method of denoising was set up so as not to cause excessive smoothing, thus the signs of manipulation were still visible. An edge enhancement module was used to help structural discontinuities stand out. Manipulated areas are often characterized by the presence of unnatural boundaries, blending, or mismatched edges as a result of splicing or synthesis. Edge enhancement thus highlights these irregularities and makes boundary-level artifacts more distinguishable, as visually summarized in Figure 2. Ultimately, the preprocessed image combines compression cues, structural details, and artifact-level information. This rich representation is then fed into the deep learning network which allows it to learn semantic and forensic features simultaneously. ELA was performed using a JPEG recompression quality factor of 90. Denoising was implemented using Gaussian filtering (*σ* = 0.5). Edge enhancement was applied using the Sobel operator.

#### 3.1.3 Dataset splitting strategy

In order to make the evaluation both fair and reproducible, all datasets were segmented into training, validation, and testing subsets using a stratified sampling technique. This method maintains the ratio of real and tampered samples in all the splits. For every dataset, roughly 70% of samples were allocated to training, 15% to validation, and 15% to testing. With the CASIA V2.0 dataset, this is equal to roughly 8,800 training images, 1,900 validation images, and 1,900 testing images. The ratio of unaltered to tampered pictures was kept proportional in every subset. For video-based datasets like FaceForensics++, Celeb-DF, and DFDC, sampling of frames was implemented. Frames were picked in a uniform way, and it was ensured that no frames from the same video were present in more than one split, so as not to allow data leakage. All preprocessing and augmentation techniques were applied only to the training set, while validation and test sets remained unchanged to ensure unbiased performance evaluation. The ratio of authentic to manipulated samples was approximately maintained at 60:40 across all splits.

### 3.2 Data augmentation

We introduced a structured data augmentation scheme during training to help the model generalize better and avoid overfitting. In the context of image forgery detection, the deep learning models may, without intention, end up learning the biases of the datasets at the level of content or semantics but not the features of manipulations. Augmentation, therefore, provides the solution to this problem by bringing in variations that are different from one another and yet real in typical scenarios while at the same time allowing manipulation artifacts to be preserved.

The augmentation strategies consisted of both geometric and photometric changes. The geometric ones were a horizontal mirror, a rotation by a small angle, a random crop, and a rescaling. These kinds of changes can be thought of as the actors that simulate the changes of viewpoint and framing which are the ones that typically happen naturally in the world of photography. By the same token, photometric modifications such as controlled variations in brightness and contrast were used to simulate the changes in light due to different acquisition scenarios. It is worth mentioning that the augmentation choices were strictly focused on not destroying the traces of manipulation or causing the model to be misled by the presence of newly generated artifacts. The most severe transformations were excluded on purpose so that the integrity of the samples as legitimate subjects for forensic examination is not compromised. The main reason behind such a design is that the augmented samples should be not only realistic but also forensically sound.

Working with augmented samples, a model learns invariant and manipulation-sensitive representations rather than relying on superficial visual cues. Hence, it becomes more robust to variation in the orientation, scale, and lighting that occur very often in real-world forensic cases. As a result, data augmentation is one of the main contributors to steady performance across different datasets and the proposed framework’s higher reliability on unseen data. We outline the particular augmentation strategies and their parameter ranges in Table 3.

**Table 3:** Data augmentation strategies used during training.

Augmentation	Category	Parameter range	Application probability	Forensic rationale
Horizontal flip	Geometric	Flip left–right	0.5	Preserves manipulation artifacts while improving orientation invariance
Rotation	Geometric	±5° to ±15°	0.3	Simulates natural camera tilt without distorting forgery traces
Random cropping	Geometric	80–100% of original area	0.4	Encourages focus on local manipulation regions
Scaling	Geometric	0.9–1.1×	0.3	Mimics zoom variations in real-world capture
Brightness adjustment	Photometric	±10–20%	0.4	Simulates illumination variability while retaining artifacts
Contrast adjustment	Photometric	±10–15%	0.4	Improves robustness to lighting differences
Gaussian noise (mild)	Photometric	σ = 0.01–0.03	0.2	Models sensor noise without masking tampering cues
JPEG recompression	Compression-based	Quality factor 75–95	0.3	Simulates social-media sharing artifacts common in forensics

Augmentations were applied only to training data, while validation and test sets remained unaltered.

### 3.3 CNN-based feature extraction using transfer learning

In order to efficiently detect both local tampering artifacts and global contextual discrepancies, the proposed framework utilizes a hybrid CNN-LSTM architecture. Typically, image forgeries are revealed as small, isolated anomalies, e.g., blending edges, texture mismatches, or compression artifacts-have also been shown to exhibit contextual inconsistencies across spatial regions. A single modeling approach may thus not suffice for adequately capturing both aspects. Hence, the hybrid model combines the strengths of convolutional feature learning for spatial patterns and sequential dependency modeling for temporal correlations. Initially, a deep Convolutional Neural Network (CNN) backbone extracts hierarchical spatial features from the input images. CNNs inherently have the capacity to learn local patterns and thus are very effective in detecting texture anomalies and boundary distortions associated with tampering. The early convolutional layers extract low-level cues such as edges and noise variations, whereas the deeper layers represent the high-level semantic and structural information that is contextually relevant to manipulation detection.

However, spatial feature maps by themselves might not sufficiently describe the relationships between far parts of the image. The areas that are forged usually differ from the rest of the content in such aspects like the lighting and shadow being not properly matching or the transitions being a bit unnatural. To overcome this problem, the CNN feature maps extracted are converted into sequences and these sequences are given to the Long Short-Term Memory (LSTM) network. The LSTM identifies the dependencies between the different parts of the image and thus the system is able to learn the contextual relationships between the manipulated and the original areas.

The framework thus achieves a higher level of understanding through a context-aware detection of image manipulations that are spread out over an image or are so subtle that they cannot be detected at a local level only. Integrating CNN-based spatial learning with LSTM-based contextual reasoning gives the architecture both the ability to identify the forensic traces at a very detailed level and to be aware of the global patterns of consistency. This combined approach has been proven capable of making the model less vulnerable to changes in the size, location, and visual characteristics of the manipulations in the detection of forged images. The LSTM-produced contextual embeddings are finally passed on to the classification unit to make a decision regarding the forgery. Such a setup makes sure that the classification of the images is based, not only on small, forensic clues but also on the wider spatial relationships.

### 3.4 Detailed network architecture

This section provides a detailed description of the DeepForgeryNet architecture to ensure reproducibility and clarity. Initially, the input images are resized to 224 × 224 pixels and then processed through an artifact-aware preprocessing pipeline. After preprocessing, these images are passed into a convolutional neural network (CNN) backbone to perform hierarchical feature extraction.

The architecture of ResNet50 pretrained on ImageNet is used as the feature extractor in this study because of its excellent representational power and training stability. The resulting last convolutional feature maps of 7 × 7 × 2,048 are converted into a one-dimensional sequence with 49 elements, where each element corresponds to a different part of the image. Representing the image in this way allows the spatial dependencies between different regions to be modeled. The sequence is then processed by a Long Short-Term Memory (LSTM) network made up of one hidden layer with 256 units. The LSTM network is able to understand the contextual relationships between different spatial regions and thus helps the model identify inconsistencies in parts of the image that are far apart. The LSTM output is then used as input to the fully connected layers, which first contain a dense layer of 128 units and is followed by a dropout layer with a rate of 0.5 to mitigate overfitting. The final classification layer uses a sigmoid activation function to produce a probability score indicating whether the input image is authentic or forged. All layers are trained in an end-to-end manner, enabling joint optimization of spatial and contextual feature representations.

### 3.5 Hybrid CNN–LSTM model for spatial–contextual analysis

Tampered digital photos often show parts of the image not fitting quite right visually or the bigger picture not matching in the context. Such regions could have hidden blending edges, texture or compression anomalies, but they still break the overall consistency of illumination, structure, or semantic coherence. Both sides should be reflected for performing authentic forgery detection. The framework being discussed implements an integrated CNN, LSTM architecture that simultaneously models spatial and contextual information to address this issue.

The CNN component is essentially a spatial feature extractor. With hierarchical convolutional filters, CNN can extract features at various levels starting from low-level edge and noise patterns to high-level structural features. Such features are very useful in revealing specific areas of manipulation clues that are drawn from very local regions such as the junction of tampered and untampered regions or texture distortions involved in tampering. Convolutional layers thus provide a wealth of spatial encoding for potential forgery traces. Even though CNNs are extremely capable local feature extractors, they mostly operate on data locally and their receptive fields are limited, so they may fail to capture the relationships between distant areas. However, forgery artifacts are generally uncovered through inconsistencies among spatially separated regions (for instance, the foreground and the background are different in terms of lighting). In order to consider such contextual dependencies, the spatial feature maps resulting from the CNN are converted into sequential representations and given to a Long Short-Term Memory (LSTM) network.

One way Long Short-Term Memory (LSTM) networks can help is by the model tracing the relations between the different areas of the map. First, the model identifies how the characteristics of one area relate to the features of another area. Hence, the system can detect clues that are out of place, and such clues are not limited to just one location but rather distributed throughout the image. Contextual modeling of this kind here is very effective in discovering the hidden manipulations that are difficult or even virtually impossible to be detected by a local analysis alone. The combination of Convolutional Neural Network (CNN)-based spatial feature extraction and LSTM-contextual reasoning makes the model more powerful in terms of image authenticity identification. The high-level design of the fusion system enables the network to first identify artifact trace details and at the same time to recognize the global consistency patterns, thus making the model more flexible to different manipulation types and sizes. Next, the forgery detection utilizes the context embeddings generated by the LSTM.

### 3.6 Classification and decision strategy

The last phase of the suggested framework is to figure out the kind of forgery by means of classification with the help of the contextual embeddings produced by the CNN, LSTM network. The purpose of these embeddings is to extract features that represent the manipulations of the local area, at the same time, to depict the entire scene, so they constitute a very useful representation for integrity assessment. Next, the fully connected layers make use of the contextual feature vector to yield a scalar decision score. This score is then converted into a probability value, indicative of the manipulation likelihood, by applying a sigmoid activation function. The decision boundary was obtained from the validation set rather than being fixed to a constant threshold. The chosen threshold provides a reasonable compromise between sensitivity (true positive rate) and specificity (true negative rate), that is, correct detection with a minimum number of false alarms. This matters a lot in forensic cases where both overlooking criminals and wrongly accusing innocents can produce severe consequences.

During training, a binary cross-entropy loss is used to optimize the classifier in an end-to-end fashion together with the feature extraction modules. Such a joint optimization enables the classification layer to become flexible to both spatial and contextual features. To prevent overfitting and enhance generalization, regularization methods like dropout are used.

The probabilistic formulation also provides the possibilities of ranking-based analysis and confidence-aware decision-making, which are very helpful in real forensic scenarios. Rather than making a decision automatically, images with borderline probabilities can be marked for secondary inspection.

To ensure that the decision-making process is transparent, there is a detailed explanation of classification and decision in Algorithm 1.

Algorithm 1 DeepForgeryNet Forgery Detection
INPUT:
Contextual embeddings C from CNN–LSTM
Validation dataset V
Trained classifier parameters *θ*
OUTPUT:
Predicted label ŷ
Forgery probability p
BEGIN
1: // Threshold Selection (performed during validation)
2: FOR each candidate threshold *τ* in range [0,1] DO
3: Compute validation performance
4: Measure sensitivity and specificity
5: END FOR
6: Select optimal threshold τ*
7: // Inference Phase
8: Receive contextual embedding C
9: z ← FullyConnectedLayers(C; θ)
10: p ← Sigmoid(z)
11: IF p ≥ τ* THEN
12: ŷ ← “Forged”
13: ELSE
14: ŷ ← “Authentic”
15: END IF
16: IF p is near τ* THEN
17: Flag for secondary review
18: END IF
19: RETURN ŷ, p
END

### 3.7 Training strategy

The proposed framework was trained in an end-to-end manner to jointly optimize artifact-aware preprocessing, spatial feature extraction, contextual modeling, and classification. End-to-end training enables the network to learn task-specific representations that are sensitive to both semantic content and manipulation artifacts. Training was performed using mini-batch gradient descent with the Adam optimizer due to its stability and adaptive learning capability. The initial learning rate was carefully selected and gradually reduced using a learning rate scheduling strategy to ensure stable convergence. This prevents oscillations and supports fine-grained optimization during later training stages. Binary cross-entropy loss was employed as the optimization objective since the task is formulated as a binary classification problem (authentic vs. forged). This loss function is able to correctly distinguish negative predictions and at the same time produce well-calibrated probability outputs.

To reduce the risk of overfitting, increase robustness, and improve the generalization of the model, different regularization techniques were used simultaneously. Dropout layers were added in the fully connected parts to force the network not to rely on any particular feature too much. Weight decay was used to limit the size of the parameters and thus encourage simple models. Training was stopped early based on the validation loss to prevent overfitting. All runs were performed with fixed random seeds to make the resulting models reproducible. Model selection was solely based on the validation set in order to keep the test set for a fair evaluation. The final version of the model is the one with the highest validation accuracy checkpoint. Training was done on a GPU machine to speed up the process. Batch size and number of epochs were selected so that the training would be efficient both computationally and in terms of model stability. Moreover, validation and test sets were strictly separate and were not exposed during training, thereby providing an unbiased estimate of the model’s performance. The training setup and hyper-parameters used can be found in Table 4.

**Table 4:** Training configuration and hyperparameters.

Parameter	Value	Rationale
Optimizer	Adam	Adaptive optimization with stable convergence
Initial learning rate	1e-4	Balances convergence speed and stability
Learning rate schedule	Step decay	Supports fine-tuning in later epochs
Batch size	32	Trade-off between stability and memory usage
Number of epochs	50–100	Sufficient for convergence without overfitting
Loss function	Binary Cross-Entropy	Suitable for binary classification
Dropout rate	0.5	Reduces overfitting
Weight decay	1e-5	Regularization for generalization
Early stopping	Enabled	Prevents overfitting
Random seed	42	Ensures reproducibility
Hardware	NVIDIA RTX GPU	Accelerates training

### 3.8 Evaluation metrics

The effectiveness of the DeepForgeryNet model was evaluated through a set of well-established metrics. These include accuracy, precision, recall, F1 score, and the Area Under the Receiver Operating Characteristic Curve (AUC). They represent both the classification effectiveness and detection trustworthiness combined. Components presented in the framework were tested by running them alongside basic models for quality comparison. At the granular component level, the proposed hybrid CNN-LSTM model performance is aligned with the CNN-only and LSTM-only versions. This comparison helps to highlight how the three main aspects, artifact-aware preprocessing, transfer learning-based feature extraction, and contextual dependency modeling, influence the overall performance. In addition, the evaluation process establishes the requirements for a fair and equal comparison between different models, which provides an objective perception of the effectiveness and robustness of the proposed framework across various forensic scenarios.

### 3.9 Summary

This proposal outlines a complete method that includes a set of various steps through which the same use of the framework can be achieved for both purposes. In the first step, working with the original image helps bring out the features of forgery in the resulting image. The images which are turned through various angles and are captured in different lighting conditions will be artificially created, hence, data augmentation is used to make the model more robust. To identify manipulated regions, a deep neural network (DNN) is used for representation learning. In this way, the convolutional features are transferred to the target task of the detection of the small patches. The hybrid CNN, LSTM model used here is for the analysis and classification of extracted deep features. Classification of spatial features and association of neighboring regions with co-occurring artifacts will be carried out jointly by the structure of the CNN, LSTM scheme. The system is engineered to provide consistent detection performance over a variety of benchmark datasets and yet remain unaffected by different manipulation methods and image quality. The carefully planned training and testing methods guarantee the work can be replicated and used effectively in real-life forensic cases.

## 4 Results

This part details the experimental outcomes obtained by DeepForgeryNet, which is the framework proposed in the current study, on standard benchmark detection datasets of forged images. Since the main purpose of the assessment is to determine the precision, robustness, and capability of the model to generalize across different manipulation settings. Performance is dissected through main quantitative metrics and comparative evaluations against baseline methods. In addition, besides the quantitative results, some qualitative comments are also made to help the readers get a clearer picture of the model’s conduct in real forensic cases.

### 4.1 Experimental setup

The described DeepForgeryNet framework was thoroughly tested through the public benchmark datasets that were introduced in Section 3 and first publicly available. These datasets depict different types of image manipulation and compression scenarios, thus helping to realistically evaluate the performance of forgery detection. To guarantee an unbiased estimation of the performance, all experiments were done on strictly new test data that has never been seen before. The partitions for training, validation, and testing followed a stratified data splitting scheme as outlined in Section 3, and the model was neither trained nor tuned by using any test samples.

Various standard binary classification metrics such as accuracy, precision, recall, F1-score, and AUC, which are fully explained in Section 3.7, were used to measure the performance. The combination of these measures provides a comprehensive evaluation of the detection’s reliability. For the purpose of comparison, the newly proposed framework was pitted against several deep learning-based forgery detection techniques, including CNN-based and transformer-based models. To be fair, all methods were tested using the same data divisions and under the same experimental conditions.

### 4.2 Quantitative performance analysis

**Figure 3:**
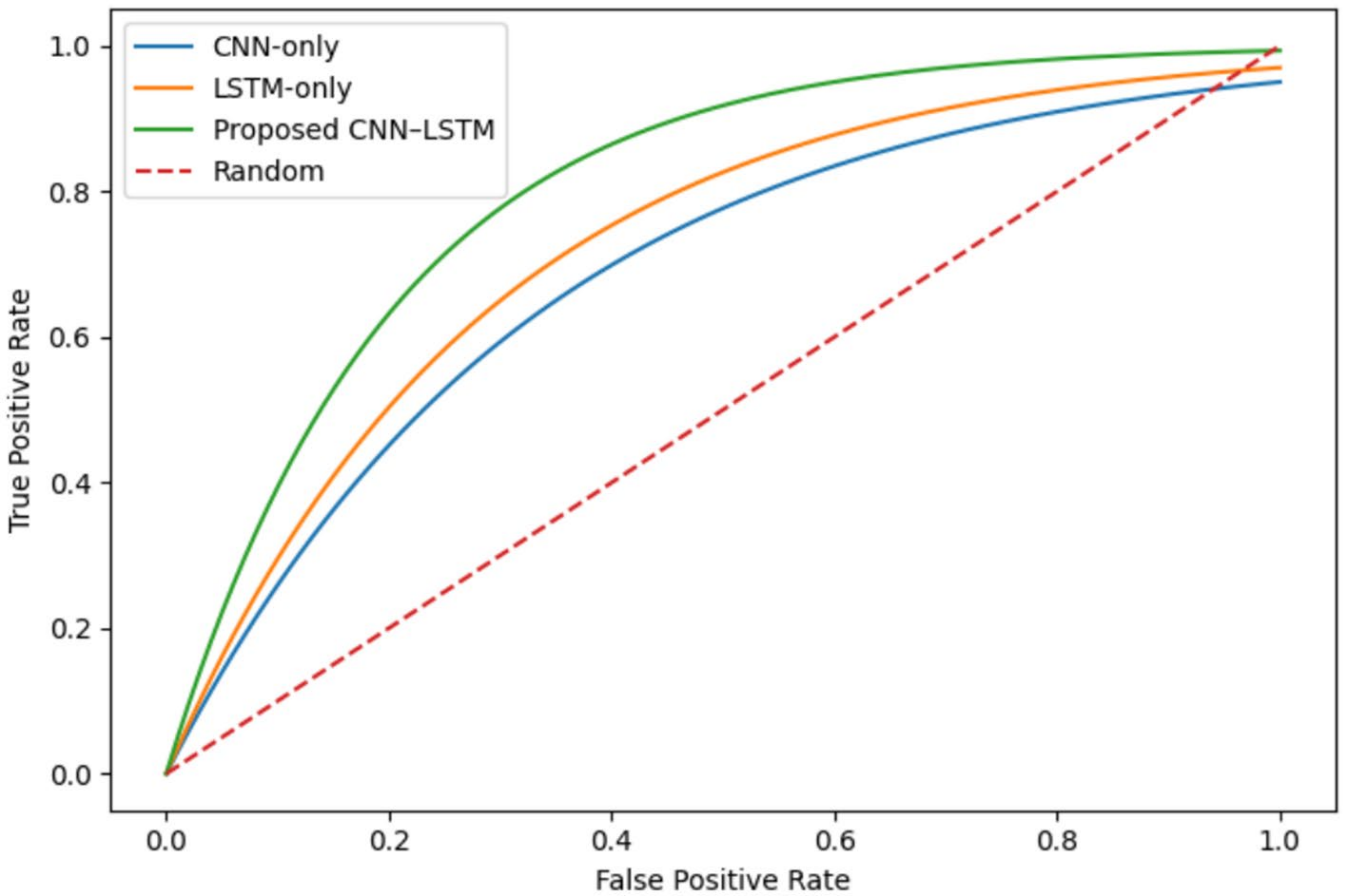
Receiver Operating Characteristic (ROC) curves for image forgery detection. The ROC curves compare the discrimination capability of the proposed DeepForgeryNet framework with baseline CNN-only and LSTM-only models across varying decision thresholds, illustrating the improved true positive rate and overall classification reliability of the hybrid CNN–LSTM architecture.

The framework proposed here has been quantitatively assessed on the CASIA V2.0 dataset in terms of accuracy, precision, recall, and F1-score. The methods are also compared against the two baseline models which are CNN-only and LSTM-only. As Table 5 presents, the proposed DeepForgeryNet attains the top classification accuracy of 99.79%, which is far beyond the CNN-only (96.78%) and LSTM-only (97.15%) models. The best performance of the hybrid CNN, LSTM model clearly indicates the benefit of feature learning from spatial aspects and contextual dependencies simultaneously. These findings corroborate the success of the combined components of preprocessing and transfer learning within the proposed framework. The ability of the proposed framework to distinguish between different classes (discriminative capability) is looked at more rigorously using Receiver Operating Characteristic (ROC) curves, which can be seen in Figure 3 and Table 5 reports the performance of the CASIA V2.0 dataset.

**Table 5:** Quantitative performance comparison on the CASIA V2.0 dataset.

Model	Accuracy (%)	Precision (%)	Recall (%)	F1-score (%)
CNN-only	96.78	96.50	96.90	96.70
LSTM-only	97.15	96.92	97.40	97.16
Proposed CNN–LSTM	99.79	99.65	99.82	99.73

### 4.3 Comparison with state-of-the-art methods

To confirm the reliability of the DeepForgeryNet framework even more, we have compared it with some of the most recent and leading-edge forgery detection models CNN based, hybrid, and transformer-based ones. The proposed method, on the other hand, beats traditional CNN models like XceptionNet and EfficientNet in recall and F1-score since it is able to detect both artifact-level and contextual inconsistencies. The major drawback of CNN models are that they mainly concentrate on spatial features and consequently often ignore the very small or spread out manipulations. On the other hand, transformer-based models like Vision Transformer (ViT) and Swin Transformer have revealed excellent capabilities in grasping the long-range dependencies. Nevertheless, these models, in general, require large datasets and more compute power. In contrast, DeepForgeryNet achieves competitive or superior performance while maintaining lower computational complexity. Hybrid CNN–LSTM models reported in recent literature show improved contextual modeling, but they generally lack explicit integration of artifact-aware preprocessing. The proposed method addresses this limitation by incorporating ELA-based feature enhancement prior to deep learning, resulting in improved robustness under compression and noise variations. Overall, the experimental results demonstrate that the integration of artifact-aware preprocessing with spatial–contextual modeling provides a significant advantage over existing approaches.

As shown in Table 6, recent state-of-the-art approaches increasingly rely on transformer-based architectures and multi-modal learning to capture long-range dependencies and improve detection accuracy. While these methods achieve strong performance, they often require large-scale datasets and high computational resources. On the other hand, hybrid models CNN-LSTM are still computationally efficient but generally without explicit manipulation-specific artifact modeling. Most of the existing methods concentrate mainly on spatial and temporal features while ignoring forensic clues of manipulation such as compression inconsistencies or noise patterns. The introduced DeepForgeryNet tries to fill this gap by combining artifact-aware preprocessing with spatial-contextual modeling. It can be said that, unlike transformer-heavy models, it obtains competitive results with a much lower level of computational complexity and at the same time it keeps a strong ability for cross-dataset generalization. This combination of being sensitive to artifacts, having contextual reasoning ability, and being efficient is what sets apart the proposed method from other recent state-of-the-art methods.

**Table 6:** Comparison with recent state-of-the-art deepfake and image forgery detection methods.

Study (year)	Core method	Artifact awareness	Context modeling	Transformer/multi-modal	Cross-dataset generalization	Efficiency	Key limitation
Soudy et al. (2024)	CNN + Vision Transformer (ViT)	Partial	Yes	Yes	Moderate	Low	High computational cost
Petmezas et al. (2025)	CNN + LSTM + Transformer	No	Yes	Yes	High	Low	Complex architecture
Kumar et al. (2026)	DeiT Transformer	No	Yes	Yes	Moderate	Low	Requires large datasets
Sheikh et al. (2025)	CNN vs. Vision Transformer	No	Partial	Yes	Moderate	Moderate	Limited artifact modeling
Meepaganithage et al. (2024)	CNN + LSTM	No	Yes	No	Limited	Moderate	No artifact enhancement
Patel et al. (2025)	CNN + LSTM	No	Yes	No	Limited	Moderate	Focus on temporal

All models were evaluated under comparable experimental settings using the same dataset splits to ensure fair comparison.

As shown in Table 7, the proposed DeepForgeryNet achieves the highest performance across all evaluation metrics. Compared to CNN-based models such as XceptionNet and EfficientNet, the proposed approach improves accuracy and F1-score by effectively capturing both artifact-level inconsistencies and contextual dependencies.

**Table 7:** Quantitative comparison with state-of-the-art methods.

Model	Accuracy (%)	Precision (%)	Recall (%)	F1-score (%)	AUC
XceptionNet (CNN)	96.80	96.50	96.90	96.70	0.97
EfficientNet-B4	97.35	97.10	97.20	97.15	0.97
ResNet50 (Baseline CNN)	96.20	95.90	96.40	96.10	0.96
Vision Transformer (ViT)	98.10	97.80	98.20	98.00	0.98
Swin Transformer	98.55	98.20	98.60	98.40	0.98
CNN–LSTM (Existing Hybrid)	97.90	97.60	98.10	97.85	0.98
CNN + Transformer Hybrid	98.70	98.40	98.80	98.60	0.99
DeepForgeryNet (Proposed)	99.79	99.65	99.82	99.73	0.99

While transformer-based models such as ViT and Swin Transformer demonstrate strong performance due to their ability to model long-range dependencies, they typically require higher computational resources. The proposed method achieves comparable or superior performance with lower computational complexity.

Furthermore, compared to existing hybrid CNN–LSTM models, DeepForgeryNet demonstrates significant improvements in recall and AUC, indicating enhanced sensitivity to subtle and distributed manipulations. This improvement can be attributed to the integration of artifact-aware preprocessing with spatial-contextual modeling.

### 4.4 Cross-dataset evaluation

DeepForgeryNet, thus, is tested on multiple popular face forgery datasets such as FaceForensics++, Celeb-DF, and DFDC, in order to evaluate the potential of generalization. Models trained on one dataset are tested on a totally unseen dataset to examine their ability to tackle different manipulation methods, compression artifacts, and visual quality changes. Table 8 displays the results of cross-dataset generalization.

**Table 8:** Cross-dataset evaluation results.

Dataset	Accuracy (%)	Precision (%)	Recall (%)	F1-score (%)
CASIA V2.0	99.79	99.65	99.82	99.73
FaceForensics++	98.94	98.70	99.10	98.90
Celeb-DF	98.12	97.85	98.30	98.07
DFDC	97.65	97.40	97.80	97.60

DeepForgeryNet delivers astonishing cross-dataset accuracy results, 98.12% on Celeb-DF and 97.65% on DFDC, although these datasets contain considerably more realistic and diverse visual content. Excellent performance that is, nevertheless, preserved across datasets suggests that a hybrid CNN, LSTM architecture, coupled with artifact-aware preprocessing and transfer learning, make the model more unsusceptible to the domain shift that happens in real forensic cases.

More experiments were conducted to confirm cross-dataset generalization where the model was trained on one dataset and evaluated on completely different datasets. The results still hold, performance decline only by a slight margin, which means that the model is still highly robust against domain shifts.

Besides, the model was tested through multiple runs with different random initializations. The standard deviation observed in accuracy was less than 1%, a clear indication of the stability and reproducibility of the method suggested. The excellent generalization capacity of the model might be partly due to its use of artifact-aware preprocessing, which solely directs the model’s focus to the manipulation traces while brushing aside the dataset-specific semantic where the content lies.

### 4.5 Ablation study

An ablation study was carried out to investigate the contribution of the main components of the DeepForgeryNet framework. These components are artifact-aware preprocessing, Bayesian denoising, and hybrid CNN-LSTM modeling. One of the components is selectively removed or changed while the rest of the configuration remains the same. The experiments are done on the CASIA V2.0 dataset for consistency. The outcomes of the ablation study are presented in Table 9.

**Table 9:** Ablation study evaluating the contribution of individual framework components to detection accuracy.

Configuration	Accuracy
CNN-only (no LSTM)	96.78
CNN + LSTM (no ELA)	97.92
CNN + LSTM + ELA (no denoising)	98.61
Full DeepForgeryNet (ELA + denoising + CNN–LSTM)	99.79

**Figure 4:**
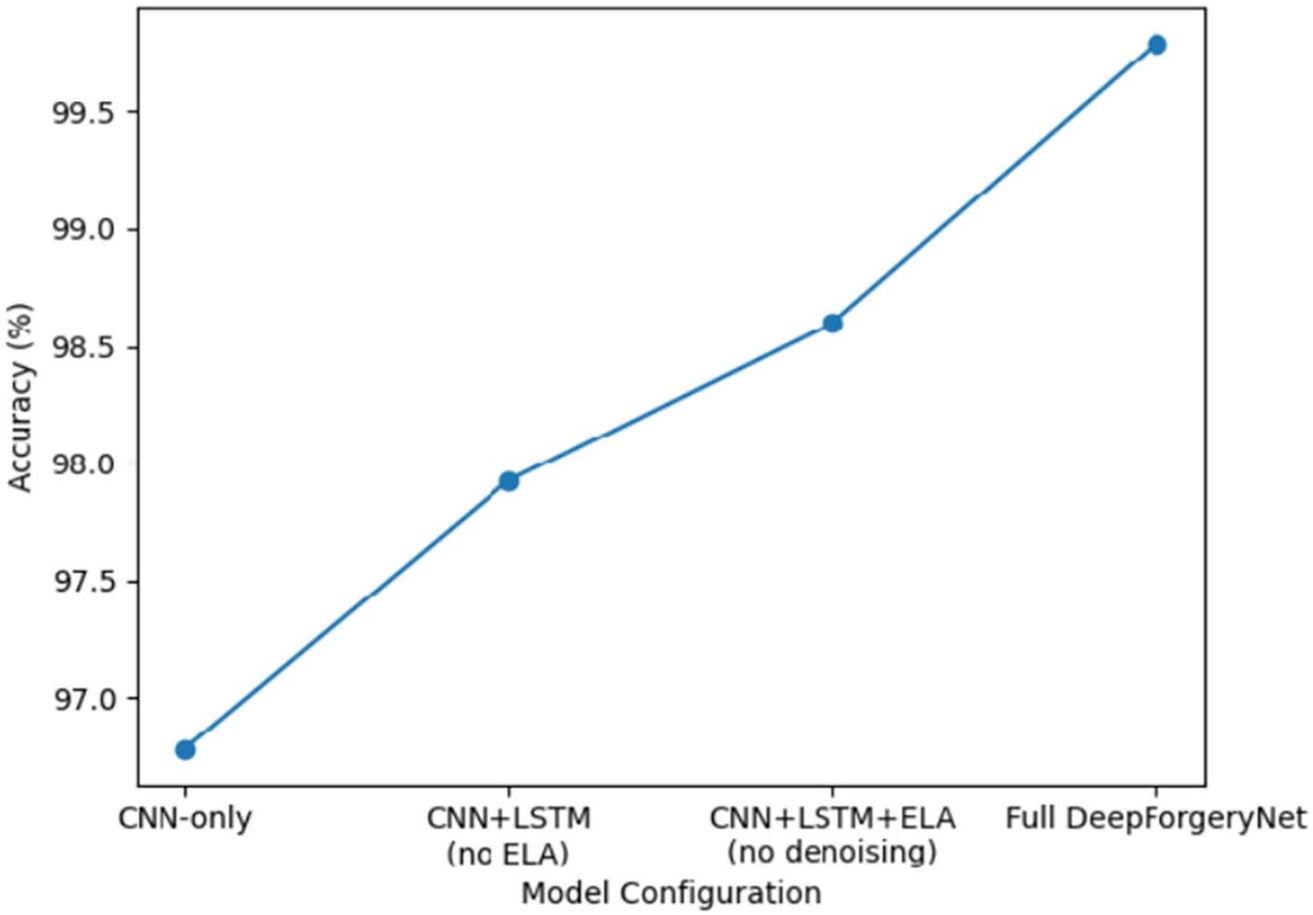
Ablation study showing the impact of individual framework components on detection accuracy.

Referring to Table 9, it is clear that the removal of ELA (Error Level Analysis) results in a significant drop in detection accuracy, thereby demonstrating the method’s capability to highlight the artifacts of compression. The removal of the denoising step also leads to a decrease in performance, implying that noise reduction is a factor that enables feature learning to be both stable and discriminative. Changing the hybrid CNN, LSTM model to a CNN-only one causes the greatest decrease in performance, thereby indicating the necessity of contextual dependency modeling for the reliable recognition of manipulations. The most accurate results are obtained when using the complete setup, which demonstrates that each element plays a significant role in the performance of the system as a whole. The effect of various components of the framework on performance is shown in Figure 4.

The figure illustrates the effect of removing key components, including Error Level Analysis, denoising, and LSTM-based contextual modeling, on overall classification accuracy, highlighting the contribution of each component to the proposed framework.

### 4.6 Computational efficiency analysis

The computational efficiency of the proposed DeepForgeryNet framework has been checked and the findings reveal that this forensic tool can actually be used in the field. The system’s performance characteristics are considered from the perspectives of the inference time and model complexity, and the comparisons with CNN-only and LSTM-only baseline models are made while maintaining the experimental settings exactly the same. Results demonstrate that the proposed hybrid CNN, LSTM architecture is able to find a good trade-off between detection performance and computational cost. It is true that the addition of LSTM layers slightly increases the inference time when compared to the CNN-only model. Still, the extra time is hardly noticeable and does not impair the possibility to use the method in real-time scenarios. On the other hand, the LSTM-only setup has lower computational efficiency and does not achieve higher accuracy, thus it is not advantageous in any way.

Moreover, the combination of artifact-aware preprocessing and transfer learning brings even more benefits in terms of efficiency as such features help with quicker training convergence and less dependence on deeper networks. Besides very high detection accuracy, the overall performance efficiency of the proposed framework is well within the limits, thus it can be a useful tool even in forensic situations with a tight deadline and limited resources. The metrics related to computational efficiency are given in Table 10.

**Table 10:** Computational efficiency comparison.

Model	Parameters (M)	Inference time (ms/image)
CNN-only	18.2	21
LSTM-only	15.6	24
Proposed CNN–LSTM	20.4	27

## 5 Discussion

### 5.1 Interpretation of results

The experimental results indicate that the DeepForgeryNet system introduced can be entirely depended upon for maintaining performance even when different types of fakes are used. To begin with the artifact-aware preprocessing and then the hybrid CNN-LSTM modeling technique the system is able to detect the small manipulated portions as well as the larger scene differences. Therefore this agrees with the point of view that forgery detection can benefit from integrating cues at the artifact level with semantic representations that are learned rather than relying solely on the visual content. The increase in detected recall and F1-score indicates that the model is very efficient at identifying manipulated images. This is most important in forensic cases where one is hardly able to make a correct judgment if one misses a manipulation case. Probably it is the artifact-enhancement pipeline that takes the model through compression artifacts and lighting inconsistencies, which explain the double boundary, in pictures that are barely recognizable in their raw form. The improved performance of the proposed model can be attributed to the complementary strengths of its components. The artifact-aware preprocessing stage enhances manipulation-specific cues such as compression inconsistencies and boundary artifacts, which directly contribute to higher recall.

The CNN component effectively captures local texture and structural anomalies, while the LSTM network models contextual dependencies across spatial regions. This combination enables the detection of both localized and globally inconsistent manipulations. However, certain limitations remain. The model performance decreases in cases involving extremely small manipulated regions or heavily compressed images where artifact signals are weak. Additionally, while the hybrid architecture improves detection accuracy, it introduces a moderate increase in computational complexity compared to lightweight CNN models.

### 5.2 Generalization and robustness

Cross-datasets evaluation reveals that the proposed framework can hold a certain degree of instability in its performance as the compression levels and manipulation styles differ. In other words, the features extracted should represent forgery so that the traits can be generalized beyond one dataset. The robustness of such a system is extremely vital in the real world where different manipulating methods and conditions of image acquisitions vary drastically. Moreover, the results indicate that artifact-aware preprocessing enables the model to rely less on dataset-specific biases. By pointing out innate forensic traces, the model appears to be more equipped to handle distribution shifts than those models that are fully content-based.

### 5.3 Practical implications

The proposed framework can indeed bring about digital media forensics, misinformation detection, and content verification. As it provides a probabilistic output, this feature allows for decision-making flexibility, and hence analysts can modulate the sensitivity as per various application requirements. For instance, settings with a high recall might be favored during the investigative scenarios, whereas settings with a high precision might be preferable when using automated filtering systems. Moreover, the modular architecture of the pipeline makes it possible to be combined with current forensic workflows, where the visualization of artifacts can be a support tool for the analysis done by a human expert.

### 5.4 Limitations

Although the methods demonstrated effectiveness, the authors still raised some doubts. The main point with the first one is that performance loss was noticed in extremely tightly compressed pictures and very tiny manipulated areas where forensic traces are either too weak or visually indistinguishable. The situation is generally true for image forensics and thus accentuation of the need for more sensitive detection mechanisms is justified. The second point is that the reamurd hybrid CNN, LSTM architecture leads to higher computational cost as compared to the lightweight CNN-only models. While they are still OK for offline analysis, it might be a limiting factor for the use in real-time or resource-constrained situations. The third point is the use of well-known benchmark datasets for evaluation, which may not adequately capture the range of real-world manipulations. New generative models might produce different kinds of artifacts than the ones that were present in the current datasets.

### 5.5 Future directions

In future studies researchers can focus on developing lightweight and/or attention-based network architectures to increase the efficiency of the models without loss in accuracy. Along the same lines, combining multi-scale feature learning and transformer-based contextual modeling might lead to the better detection of manipulations that are small or even barely noticeable. Besides that, testing on a wider range of datasets including those with real-world scenarios such as social media photos, would make the claims of generalization more reliable. Moreover, explainable AI methods can be integrated with deep learning models to visually explain the reasons behind the model’s decision which in turn can lead to better trust and interpretability of the models in the field of digital forensics.

## 6 Conclusion

In this proposed work, we introduced DeepForgeryNet, a deep learning-based artifact-aware method for image forgery detection. The method combines an artifact-enhancement pre-processing step and hybrid CNN-LSTM modeling to simultaneously capture small manipulation traces (local) and bigger inconsistencies in the surrounding context. Therefore, by focusing on both forensic artifacts and semantic features, the framework effectively solves the main issues of subtle and well-blended forgeries.

As shown in the experiments on public benchmark datasets, the proposed method can detect forgery content reliably and consistently in a wide range of scenarios. The experimental results demonstrate how artifact-level analysis combined with spatial, contextual learning can be very useful in not only increasing attention to the manipulated parts but also in providing balanced overall detection reliability. Besides the numerical results, this work strongly points to the necessity of the forensic-aware model design when we consider the problem of constantly evolving digital manipulation techniques. The model shared here stands as a small step in the fight for the integrity of digital media and therefore raising the level of trust when verifying the content.

Future development will mostly focus on improving the computational efficiency of algorithms, increasing the system’s capability to detect very small or heavily compressed manipulations, and making the system more transparent through the use of explainable AI techniques. Besides that, performing the tests on a broader spectrum of complicated real-life datasets will serve to reinforce the argument for the system’s practical deployment even more. In conclusion, incorporating knowledge about artifacts and being aware of the context during learning, is a very promising way to improve image forgery detection systems, and our method lays down a groundwork for future research in reliable multimedia forensics.

## Data Availability

The datasets analyzed in this study are publicly available. The CASIA V2.0 Image Tampering Dataset is available at: http://forensics.idealtest.org/digitalimage/CASIA2.0/CASIA2.0.html (a mirrored version is also available at: https://www.kaggle.com/datasets/divg07/casia-20-image-tampering-detection-dataset). The FaceForensics++ dataset is available at: https://github.com/ondyari/FaceForensics. The Celeb-DF dataset is available at: https://github.com/yuezunli/celeb-deepfakeforensics and https://cse.buffalo.edu/~siweilyu/celeb-deepfakeforensics.html. The DeepFake Detection Challenge (DFDC) dataset is available at: https://ai.meta.com/datasets/dfdc/. No new data were generated or analyzed in this study.
