# Supplementary material for: DeepForgeryNet: a hybrid CNN–LSTM and transfer learning framework for robust image forgery and deepfake detection
Source: Front Artif Intell. 2026 May 8;9:1810912. doi: 10.3389/frai.2026.1810912 (PMC13194494; doi:10.3389/frai.2026.1810912)
Supplement: Supplementary file 1 [file Supplementary_file_1.docx]

Prompts used to create images for Figure 1 and Figure 2

“A flowchart diagram of an artifact-aware image preprocessing pipeline showing input image, Error Level Analysis (ELA), denoising, PSNR validation, edge enhancement, and final output, with arrows connecting each step, using a sample dog image.”

“Illustrate a step-by-step image processing pipeline with modules for ELA, Gaussian denoising, PSNR quality assessment, and Sobel edge enhancement, with a clean academic diagram style.”

“Generate a research-style block diagram showing sequential image enhancement stages applied to a sample image (dog), including labeled modules and intermediate outputs.”
